# Arbiters of Time: The Experience of Adults Aging with Spinal Cord Injury

**DOI:** 10.3390/jal3010005

**Published:** 2023

**Authors:** Lisa Reber, Nasya S. W. Tan, Michelle A. Meade, Martin Forchheimer, Denise G. Tate, Philippa Clarke

**Affiliations:** 1 Department of Physical Medicine and Rehabilitation, University of Michigan, Ann Arbor, MI 48108, USA; 2 Center for Social Epidemiology and Population Health, Department of Epidemiology, School of Public Health, University of Michigan, Ann Arbor, MI 48109, USA; 3 Institute for Social Research, University of Michigan, Ann Arbor, MI 48104, USA

**Keywords:** spinal cord injury, disability, qualitative study, interviews, focus group, temporality, environmental factors

## Abstract

Time is a fundamental component of our lives. It is both objective, a structure outside of ourselves, and subjective, an element that is relative to the life we live and how we experience it. The disabled body must come to terms with time to understand the future impact of the injury and its progression, as well as how the injury will impose a new more accelerated aging process in the body, resulting in a compressed lifespan. The body also challenges time’s control of the body. This paper extends the literature on the study of time to the experience of adults aging with a spinal cord injury (SCI). Drawing from interviews conducted with adults with long-term SCI, it examines how their narratives about aging and the proactive management of their lives reflect their orientation toward and anticipation of the future. Recognizing that the spoken word often carries a multiplicity of meanings, it considers what participants’ words might imply about their engagement with time. The results of this study show that the process of aging is characterized by uncertainty and the expectations of functional and health decline, requiring a sense of urgency and vigilance in the face of the uncertain course of aging with SCI. Participants understood that their lifespan was compressed due to the physiological impact of accelerated aging. Knowledge of this compression made time a scarce resource. Yet, despite it being the arbiters of their futures, so too were they the arbiters of time.

## 1. Introduction

In the 1940s, life expectancy for individuals following a spinal cord injury (SCI) was a mere 18 months [1]. Today, adults injured at age 20 are expected to live into their 50s and 60s, due in part to advances in medical care and technology [2]. However, life expectancy for persons with SCI still remains significantly lower that of persons without SCI [3]. The shorter lifespan is due to the cumulative effects of living with SCI for many years [4–6]. Its physical impact depends on the age and level of injury. The higher the injury to the spinal cord, the more severe the impairment. This impairment not only impacts the ability to walk or the use one’s arms, but also affects the respiratory, cardiovascular, and gastrointestinal systems. Accelerated aging processes are indicated in multiple body systems [7–9], including the premature onset of chronic health conditions and the development and progression of secondary health conditions, such as pain, pressure ulcers, reduced lung capacity, and spasticity, as well as damage from repetitive motions, such as those caused to the rotary cuff from wheelchair use [10,11]. Acceleration is worsened through factors such as allostatic load, weathering, and the time and stress of managing a body and life impacted by SCI in a world often lacking in adequate access, accommodations, and resources.

Time is a fundamental component of our lives. It is both objective, a structure outside of ourselves, and subjective, an element that is relative to the life we live and how we experience it. Adam refers to the first as events in time, a unit, unrelated to anything else, and the latter as time in events [12]. The second is inherently cultural and closely related to context. For example, for Americans, time is often considered a commodity and, when used well, a sign of competence. Time has also been identified as a privilege and a social resource [13]. It is foundational to multiple facets of daily life, as well as longevity, and is even considered to be a social determinant of health [14], due in part to allostatic load [15] and the social “weathering” it can impart [16]. Gee et al., who examined time in the context of racism, contend that racism manifests as lost time through mechanisms such as stress and the inability to engage in health-promoting activities, which then contribute to health inequalities, reflecting its inequitable distribution [13]. This inequitable distribution of time can also apply to adults with spinal cord injury (SCI), especially for those aging with long-term SCI. Yet, to date, very little research has considered the meaning of time for this population.

In *Time and the body: Re-embodying time in disability*, Seymour examined how SCI disrupts how time is imbedded and functions in relation to events, processes, and the body [17]. Time, she explained, exerts control over the body and dictates its management; meanwhile, at the same time, the body dictates how time is used. In the first, time is disembodied. It is an independent factor, a framework within which events occur and are experienced. The disabled body must come to terms with time to understand the future impact of the injury and its progression and how the injury will impose a new more accelerated aging process in the body, resulting in a compressed lifespan. The body also challenges time’s control of the body. Seymour examined how the body integrates time by analyzing the process of re-embodying daily practical activities, such as bowel and bladder management. Nespor et al. examined how the temporal barriers created by the school system were experienced by children with physical and cognitive disabilities [18]. He showed how time is a system that is taken for granted because institutions expect all people to understand in the same way they do. For children who experience the world differently, a singular understanding of how time and space is experienced can exclude them from shared activities and engagement. Additionally, drawing from her own experiences, disability activist Miserandino illustrated to a friend through a limited number of spoons how she must ration how her time is expended through the daily tasks of living: one spoon for getting up, another (or two) for taking a shower [19]. The spoons reflect the limited amount of energy and possibilities she has available each day. Her management of them reveals the control that her body and its physical requirements have over time and how it is used.

This paper extends the literature on the study of time and the experiences of adults with disabilities. Drawing from interviews conducted with adults with long-term SCI, it examines how their narratives about aging and the proactive management of their lives reflect their orientation toward and anticipation of the future. Recognizing that the spoken word often carries a multiplicity of meanings, we consider what participants’ words might imply about their engagement with time.

## 2. Methods

This paper uses cross-sectional data collected for the qualitative component of a multi-methods study examining the subjective experiences of adults aging with SCI in relation to their surrounding social and physical environments, including the impact of barriers and facilitators (for details of the larger study see [20]). The larger study included a structured telephone survey with a convenience sample of 182 community-dwelling adults aging with SCI living in three Midwestern states (Michigan, Ohio, Indiana). The inclusion criteria required the following from the participants: (a) be 45 years of age or older; (b) have a SCI or a disease resulting in damage to the spinal cord at least 5 years prior to study enrollment; and (c) live within the community (not within an assisted-living facility or nursing home). All participants provided informed consent, and the study procedures were reviewed and approved annually by the institutions’ Institutional Review Boards. Participants received a nominal incentive for their participation. A subsample of 24 participants participated in qualitative interviews (N = 13) and focus groups (N = 11) to capture more detailed perspectives on the experience of aging with SCI. They were selected with the aim of capturing the diversity of experiences among participants with SCI in terms of gender, socioeconomic status, and urban/rural residence (see Table 1).

Coding and analyses were driven by a constructivist perspective which focuses on how participants’ perceptions and experiences are “the effect of a range of discourses operating within society” [21] (p. 81), see also [22]. This informed our analyses which drew on the work of Braun and Clarke’s [23] constructivist reflexive thematic approach (see [24]). This approach supports attending to people’s views and experiences and the examination of data that are richly detailed and nuanced. The process of analysis was iterative, and continually evolved. Braun and Clarke’s [21,23] process of data analysis guided us: familiarizing ourselves with the data; moving between the initial identification of codes and patterns in the data and the development of categories, themes, and subthemes, as well as sorting the associated narratives; and finally, naming and defining the themes (see Appendix A for methodological details and Appendix B for interview and focus group guide).

## 3. Results

Of the total 24 adults with SCI injuries who participated in a focus group or interview, 23 were included in this study. One participant was excluded because of a lack of data due to a recording error during the interview. In our analyses, we identified two overarching themes: (1) time’s control of the body and (2) the individual’s control over time.

### 3.1. Time’s Control of the Body

The first theme we identified addresses the relationship between time and the progression of SCI. Time was an independent event through which aging, and the process of disablement, occurred at more accelerated rates. Walter, aged 66, who required a power wheelchair but who could still use a walker for minimal distances, said, “Things are changing a lot faster than I expected them to [ . . . ] sort of clipping along at a faster pace than you expected.” Uncertainty about the cause of this acceleration and whether it was due to systematic inflammation, immunosuppression, and multiple organ dysfunction [25], or wear and tear through use [1], was revealed in participant comments about their injuries worsening with age. Jeffrey, a man aged 48 with tetraplegia who used a power wheelchair, said, “So, that’s a fear, you know, and that could be something with age or not even age as much as just use.” Other participants were more certain about what would cause their decline. Angela, aged 47, who used a manual wheelchair, knew that the progression of her injury “involved a reduction in lung capacity.”

Often, participant comments about aging and the progression of their injury addressed loss of physical functioning and mobility. For example, Jeffrey said:

If I got weaker, that would be a problem, because I’m pretty much like, the way I move now uses most of my strength to like get dressed and whatnot . . . It’s surprising what things are, you know, tiring, because fatigue is definitely a part of this. So I would say yeah, getting older, the biggest thing I would think of yeah, might honestly be just, you know, losing strength or something like that.

Similarly, Christopher, a man of the same age and with a similar condition to Jeffrey, said, “[I’m] worried about losing strength and, you know, function in general [ . . . ] You never know what’s coming.” Fear about these anticipated futures was evident. George, aged 58, who used a manual wheelchair, had in the first decade following his injury been in a wheelchair but still had a very high level of functioning. However, he had been reinjured and, though he was still able to use a manual chair, said, “I don’t know what ability I’m going to lose next. So I’ve got to hold on to what I can do tightly.” Furthermore, Albert, aged 47, who could still walk with the assistance of a cane and had far more functioning than the majority of participants, expressed his fear about increased impairment. He saw his future as tenuous because of the surgery his doctors recommended. He was “afraid” they would “screw it up” and he would end up like the other men in his focus group. He said, “I mean no disrespect to the gentlemen that are wheelchair-bound already, but that’s like one of my biggest fears.”

In the participants’ comments, we can see both an awareness of and concern about the loss of function that would come with accelerated aging. We can also infer from their comments that they see time as the arbiter of their futures, an independent factor that frames and delineates their lives, with implications for a compressed lifespan.

### 3.2. The Individual’s Control over Time

Although the participants showed an understanding of time as a disembodied element which their bodies had to negotiate, the participants can also be seen as attempting to control and deaccelerate time and extend their futures. Challenging time’s control of the body took the form of rejecting the limits that time was imposing on their futures or confronting the imposition of time by proactively structuring their daily lives. It is through control over the present that participants attempted to control their future and bend it to meet their circumstances.

#### 3.2.1. Rejecting Time’s Control

At certain points in their narratives, a rejection of accelerated aging and a hopeful anticipation of (or perhaps simply a desire for) an extended future is communicated. Christopher said, “I am hoping I keep getting better and better . . . . I think I should be fine.” This sense of hope could be considered resistance to a future where he would have reduced functioning and increased need. We can also identify a determination to remain optimistic. Jeffry, who had experienced small improvements in his limited motor skills in the ten years since his injury at the age of 38, said, “certain things have gotten a little better.” Knowing the extent of their injuries, we might conclude that they are in denial. However, it may be a survival mechanism or an active choice to reject the imposition of time on their bodies.

Some participants reminded us that life is complex and so too is our personhood (see [26]). Ruth, aged 61, who used a manual wheelchair, seemed unable to mentally resist the advancement of time and the challenges it brought. She described herself as being “lazy” and finding physical therapy “exhausting.” She spoke of being worn out by her daily battle:

Just, the strength it takes to do my everyday thing and you know, I threw out my shoulders for a while there and I had to do physical therapy for that and then it just wears me out so much to where I don’t want to do much else.

Yet, she also rejected a future that would, when suggested, require her to use a power wheelchair: “Don’t do that to me, no, I won’t!” Angela, too, presented the contradictory personhood that is in us all, contending that getting older required “being proactive in maintaining a healthy lifestyle”, while also knowing that if she did “everything [her] doctors wanted [her] to do regularly, [she] couldn’t hold a job.”

#### 3.2.2. Preempting the Acceleration of Time

Participants provided evidence of controlling their futures and attempting to deaccelerate the advancement of time and its impact on their bodies through their proactive efforts to maintain their health and functioning. Evidence of this can be identified in the participants’ comments about maintaining, strengthening, and protecting their bodies. Participants spoke about the steppers, ellipticals, or handcycles they used for physical exercise, or the need to go outside and move, or engaging in mental exercise. Physical therapy was central to this because, as Walter explained, it “keeps the joint pain down,” “slows the deterioration,” and “helps the muscle spasms.” Some were motivated to exercise because they could “see the difference” when they did (Taylor). Others did so because they found that for a body that sits all the time, movement had “psychological” benefits (Angela).

Functioning, however, did not always entail the ability to physically move their bodies. Max, aged 58, who was paralyzed from the neck down, said he took care of his health by “Doing what I [am] supposed to do, take care of the mind, you know, either way aging the best you can.” He explained that achieving that required one to, “Get up every morning, do a daily routine, just like everybody else. ... If you do all of that . . . your diet’s good, you have a very good chance.” Lucas, aged 65, who used a manual chair, started every morning with yoga, but also engaged in activities to maintain his mental and emotional wellbeing. He stated, “At home, [I do] wheelchair yoga. I’m doing my meditation in the morning before I even get up, and I do my exercises . . . . I try to stay consistent with that.”

In addition to engaging in exercises for the mind and body, time was also proactively decelerated, and bodies were maintained through a good diet. Sophia was 71 and required a power wheelchair and, though she needed assistance, still had use of her hands. She explained,

I’ve always been a very healthy eater. I think that has a lot to do with being healthy. The healthier you eat, the healthier your body is going to be. So, I make everything from scratch. [...] I don’t do a lot of that [eating out] because I prefer my own cooking, and yeah, I choose to eat healthy, and I find a lot of foods are not healthy.

For Jennifer, aged 54, deciding to change her diet reduced her pain, increased her energy, and improved her mood and overall quality of life. She used a power wheelchair and a manual wheelchair for exercise. She said, “For the first time, the normal aches and pains I had weren’t there.”

Maintaining their bodies also involved preempting negative health and functioning that can arise from poor hygiene, secondary conditions, and comorbidities. Jimmy and William, ages 69 and 49, respectively, spoke about the precautionary measures they took when out in public. Both feared infections. They washed their hands frequently and as William, who, unlike Jimmy, used a manual wheelchair, said, “[I use] hand sanitizer on everything I possibly can.” Others addressed how they attempted to preempt potential secondary conditions, such as pressure sores and nail care. Deborah, aged 55 and paralyzed from the neck down, was careful about the amount of time she spent in her wheelchair because of chronic pressure sores. Thus, “to keep me out of the hospital, keep my wounds under control, I spend a lot of time in bed.” Christopher had his personal care assistants check those parts of his body for pressure sores that he was unable to see:

You got to keep up on some of the things, you know, even like things as simple as nail care. And you know, if you don’t keep up on that, then you’d get an ingrown nail. . . . It’s really important, at least for me, you know, to have some routines, you know, just constantly being checked by people to make sure that everything’s good to go basically.

Deborah said her family got mad if she failed to take the necessary precautions to avoid pressure sores. She said, “Like if we go on trips and I’ll be up for two days in a row, I’ll come home, and they’ll be mad [because] I’ll be a little raw.” It was a minor inconvenience compared with her other challenges: “In light of everything else I go through, that’s nothing, you know, it’s just another daily thing to deal with.” Comorbidities were also a concern. For example, Jeffrey watched his diet because he knew that his lack of mobility and the weight gain that could come from it put him at risk for other conditions, such as heart disease. This motivated him to “do the best [he could] to stay healthy and stay mobile.”

Aging well with SCI requires vigilance because, as one participant contended, people in wheelchairs “age a little harder.” This sentiment was one that several participants agreed with. Taylor, aged 47, who used a power wheelchair during the day but was able to walk limited distances and use her manual wheelchair for exercise, said, “I can see where aging can be a struggle, but I think it’s just, uh, determination to be able to, you know, be able to do those things, and get out, and you know, and get around”. While some participants may have (at times) seemed unrealistically hopeful about their impairments, others showed they were not. Walter lived in a two-story house that was quickly becoming inaccessible. He felt the need to consider new housing options so that he could “be prepared and know what’s out there, what it’s gonna cost . . . And brace for that.” Bracing is digging in the heals. It is both standing still and movement. It is reinforcing the mind. For Walter, bracing enabled “looking into that future of the unknown” and “planning for what happens when things change.” In research on how anticipatory thinking is manifested, Klein et al. states that “anticipatory thinking is the process of imagining how unexpected events may affect plans and practices” and involves “guard[ing] against or forestall[ing] potential threats” [27] (p. 235).

The anticipation of a future “clipping along at a faster pace” and the notion that people in wheelchairs “age a little harder” prompted a desire to maintain a level of functioning into the future that motivated participants to actively care for their bodies. It required “determination” and “follow through” to maintain their bodies and carry out the mundane (yet, at times, complex) planning required to visit the doctor or go shopping. The underlying presence of proactive thinking was demonstrated when, for example, participants spoke about exercise, physical therapy, diet, and the importance of “staying consistent”, having a “routine”, and “keep[ing] up with everything”. Participants were not passive recipients of their accelerated lives. They actively fought to be the arbiters of their futures.

## 4. Discussion

From a lifespan perspective, aging is biographical time that is marked by key life stages (e.g., childhood, adolescence, early adulthood, midlife, later life) [28]. The age-graded structure of the life course is seen as a system of social roles where age is a major criterion for entry into or exit from roles over time [29–31]. For example, as people age, they generally move through the structural stages of family life, school, career trajectories, retirement, and ultimately death [32].

This normative understanding of the life course has led disability scholars, often using a crip analysis, to challenge it. The term “crip” is a reclaiming of the word “cripple,” which has historically been considered a derogatory term. Crip, write Hutcheon and Wolbring, “depicts a critical orientation to the world, a positionality, and a process by which power structures and oppressive assumptions are revealed and disrupted” [33]. Time from a crip perspective disrupts normative experiences of the life course [34] because, as Samuels writes, disabled lives involve “a wormhole of backward and forward acceleration, jerky stops and starts, tedious intervals and abrupt endings” [35] (p. 35).

Time, in relation to daily activities, is also cripped. That is, the normative understandings of time, schedules, and routines, organized according to temporal structures imposed by ableist institutions, is questioned and challenged. In industrial societies, time and money and, hence, the ability to use time efficiently and profitably are “valorized” and valued over other considerations; as a result, those who are unable to use time effectively are devalued and considered backwards, and the inequalities they produce are “rendered invisible” [36] (p. 124). Crip time, contends Samuels, “bends the clock to meet disabled bodies”. It redefines time and shapes it to a temporal framework that challenges normativity [35].

The results of this study show that the process of aging is characterized by uncertainty and expectations of functional and health decline, requiring a sense of urgency and vigilance in the face of the uncertain course of aging with SCI. Participants understood that their lifespan was compressed due to the physiological impact of accelerated aging. Knowledge of this compression made time a scarce resource. Yet, despite it being the arbiters of their futures, so too were they the arbiters of time.

### Limitations

Despite the rich data collected during this study, they were exploratory and did not focus specifically on the role of time. However, extensive discussions about the factors that impacted their lived experiences provided the space needed for their experiences with time to be identified. Another limitation is that neither of the two authors who were central to conducting the coding and analysis have a disability. However, the lead researcher has over two decades of experience conducting research on aging in people with disabilities, and the lead author has over eight years of experience researching structural inequities among marginalized populations, including three years focusing specifically on adults with disabilities.

## 5. Conclusions

Time is socially patterned, and this patterning contributes to health inequalities [13,14]. A scarcity of time can be a disadvantage by preventing adults aging with SCI from engaging in work and other activities. This scarcity is not only due to impairments that have additional time requirements. It is also due to a lack of equipment, caregivers, transportation, or other resources that can help facilitate the activities in which people with SCI engage. The lack of these resources and the inequities they lead to are not fixed. It is the result of systems that are not designed to meet the needs of people with disabilities [37,38]. Healthcare and rehabilitation practitioners should be cognizant of the challenges faced by their patients and be aware of the importance of listening to their patients’ needs with respect to aging. Future research should examine the patterning of time–space resources and their impact. In addition, limited research has examined the impact of socio-economic status on aging with a disability and, hence, how time is a privilege that is unequally distributed (see [13]). Additional research could examine how income and access to resources impacts how time as exposure and as a resource are differentially experienced by adults aging with disability. It could also examine the sociospatiality of time, including how access is provided through environmental factors, how the responsiveness of social structures is experienced, and how for individuals with disabilities, these factors and structures are time-dependent.

## Figures and Tables

**Table 1.** Participant characteristics.

Pseudonym	Age	Years since Injury	Ability/Level of Functioning ^b^	Household Income and Other Financial Resources^a^	Race	Lives with	Education
Focus Group 1							

Jimmy	69	5	Paraplegia, power w/c	Household income: $40–59k	White	Spouse/SO	BA/BS or +
William	49	16	Paraplegia, manual w/c	Household income: $25–39k	White	Alone	Some college
Stephen	57	27	Tetraplegia, able to walk without assistance	Household income: >$80k	White	Spouse/SO	HS/GED
Albert	45	24	Paraplegia, able to walk with cane	Household income: $60–79k	White	Spouse/SO and children	BA/BS or +

Focus Group 2							

Christopher	48	8	Tetraplegia, power w/c, minimal use of one hand	Household income: <$25k	White	Alone	BA/BS or +
Jeffery	48	10	Tetraplegia, power w/c	Household income: $40–59k	White	Roommates	HS/GED
John	59	5.5	Paraplegia, able to walk with assistance	Household income: >$80	White	Spouse/SO	<HS

Focus Group 3							

Joe	66	15	Paraplegia, power w/c, stand w/walker	Household income: <$25k	White	Spouse/SO	Some college
Jose	58	22	Tetraplegia, power w/c, no fine tune motor skills or ability to transfer	Household income: <$25k	White	Sibling	Some college
Samuel	57	37	Paraplegia, power w/c	Household income: <$25k	White	Alone	Some college
Michael	63	18	Tetraplegia, able to walk with cane	Household income: <$25	White	Children and grandchildren	Some college

One-on-one Interviewees							

Adam	63	10	Paraplegia, power w/c, manual w/c	Household income: $25–39k	White	Spouse/SO	Some college
Angela	47	28	Paraplegia, manual w/c	Household income: >$80k Full–time work	White	Spouse/SO	PhD
Deborah	55	25	Tetraplegia, power w/c	Household income: >$80k	White	Children and grandchildren	HS/GED
George	58	38	Paraplegia, manual w/c	Household income: $25–39k		Alone	BA/BS or +
Jennifer	54	26	Paraplegia, power w/c	Household income: not given Estimated: >$80k	White	Spouse/SO	Some college
Lucas	65	26	Paraplegia, power w/c w/assist	Household income: $60–79k	African American	Spouse/SO	HS/GED
Max	58	18	Tetraplegia, power w/c, sip and puff	Household income: <$25k No-fault insurance	White	Parents and caregiver	HS/GED
Oliver	60	27	Paraplegia, manual w/c	Household income: <$25k + Receiving severance pay	White	Spouse and children	BA/BS or +
Ruth	61	17	Paraplegia, manual w/c, walker	Household income: <$25k + Additional financial resources	White	Alone	HS/GED
Sophia	71	19	Tetraplegia, power w/c, some use of hands	Household income: <$25k No-fault insurance	White	Alone	Some college
Taylor	47	10	Tetraplegia, power w/c, manual w/c, walker for short distances	Household income: $60–79k No-fault insurance; full–time work	White	Alone	BA/BS or +
Walter	66	30	Tetraplegia, power w/c, walker for short distances	Household income: $40–59k No-fault insurance; retired	African American	Alone	BA/BS or +

Average	57	20					

a Household income is the amount declared by participants in survey conducted prior to qualitative study. Additional financial resources were indicated during interviews.

b Level of physical functioning is indicated by inclusion of assistive technology used. SO—significant other. w/c—wheelchair. HS/GED—high school diploma. No-fault insurance—Michigan’s no-fault insurance protected insured persons from being sued as a result of an automobile accident.

## Data Availability

Some of the data presented in this study are available on request from the corresponding author. The data are not publicly available due to the privacy of participants.

## Appendix A.

### Appendix A.1. Methodological Details

#### Appendix A.1.1. Study Overview

This paper uses cross-sectional data collected for the qualitative component of a multi-methods study examining the subjective experiences of adults aging with SCI in relation to their surrounding social and physical environments, including the impact of barriers and facilitators. The larger study included a structured telephone survey with a convenience sample of 182 community-dwelling adults aging with SCI living in three Midwestern states (Michigan, Ohio, Indiana). Participants were recruited from SCI rehabilitation centers, community organizations, centers for independent living, and the SCI registry and outpatient clinics at a large hospital in the Midwest. The inclusion criteria required the following from the participants: (a) be 45 years of age or older; (b) have a SCI or disease resulting in damage to the spinal cord at least 5 years prior to study enrollment; and (c) live within the community (not within an assisted-living facility or nursing home). A total of 187 participants were examined for eligibility and 5 were deemed ineligible due to residence outside the study area. All participants provided informed consent and the study procedures were reviewed and approved annually by the institutions’ Institutional Review Boards.

The quantitative survey data were collected via computer-assisted telephone interviews between January 1, 2019 and September 30, 2020. A subsample of participants was selected to participate in qualitative interviews and focus groups to capture more detailed perspectives on the experience of aging with SCI.

### Appendix A.2. Recruitment and Data Collection

Participants were selected for the qualitative component with the aim of capturing the diversity of experiences among participants with SCI in terms of gender, socioeconomic status, and urban/rural residence. Interviews were conducted in addition to focus groups for two key reasons. The first was to add in the voices of demographic groups who were underrepresented in the quantitative component, namely Black/African Americans and women. Members of our research team recommended separate focus groups because the voices of these populations are often lost in white male majority groups. Interviews were conducted instead of focus groups because we were either unable to recruit a sufficient number to form a focus group or find a time convenient to conduct them. The second reason we conducted interviews was because this setting provides the opportunity to ask participants to describe in greater detail the different features of their environments and to allow them to be more personal and reflective.

Three focus groups were conducted with 11 participants, and an additional 13 participants completed an in-depth interview. The first focus group was conducted on 19 August 2020 and consisted of four participants living in rural environments. The second was conducted on 26 August 2020 and consisted of three participants living in urban environments and of a relatively younger age (age < 55 with one exception). The third and final group was conducted on 3 September 2020 and consisted of four participants living in urban environments and of an older age (age > 55), with low SES. Participants for the in-depth interviews included seven White women, three African American men, and two Native American men. They were conducted between August and October 2020.

The focus groups and interviews were conducted over Zoom due to the COVID-19 pandemic (See Table 1). Each focus group was 60 min long and had a moderator, a comoderator, and a note-taker. The lead researcher joined a portion of the first call and the entirety of the remaining two. The groups were audio-recorded with the participants’ consent after the introductions and guidelines were read. The interviews ranged from 40–60 min. Each interview was conducted by one of two experienced qualitative research associates. The focus groups and interviews were semi-structured. Questions were the same for both. They addressed the accessibility of the participants’ lived environments, their access to healthcare and health insurance, how they adapt to their environments, and how their needs and priorities change as they aged (see Appendix B for interview and focus group guide).

All audio recordings were transcribed by a transcription service and deidentified by the research team upon receipt. One of the twenty-four interviews, an interview with a White woman, was not included in the analysis because of a recording error. The transcripts were labeled with the participants’ subject ID and a pseudonym. Transcripts were reviewed for accuracy by a research assistant prior to analysis. After deidentification, the transcripts were imported into NVivo for analysis.

### Appendix A.3. Analysis

Coding and analyses were driven by a constructivist perspective which focuses on how participants’ perceptions and experiences are “the effect of a range of discourses operating within society” [21], see also [22]. This informed our analyses, which drew on the work of Braun and Clarke’s constructivist reflexive thematic approach [23], see also [24]. This approach supports attending to people’s views and experiences and the examination of data that are richly detailed and nuanced. The process of analysis was iterative, and continually evolved. Braun and Clarke’s [21,23] process of data analysis guided us: familiarizing ourselves with the data; moving between the initial identification of codes and patterns in the data and the development of categories, themes, and subthemes, as well as sorting the associated narratives; and, finally, naming and defining the themes. Keeping in mind these essential core steps in qualitative data analysis, we decided to take a slightly different approach.

We read through the data to obtain a general sense and then explored it in greater depth in the context of wanting to understand the essence of the participants’ thinking, attitudes toward healthy aging, and the factors that contribute to it, while also attending to variation and the unexpected. This required repeatedly reexamining participant narratives and associated codes to capture a deeper understanding [39]. We began by conducting initial coding of three transcripts using NVivo. However, while we normally use NVivo to code all of our transcripts, we felt a different approach was needed. Sometimes, alternative approaches can help researchers see data in a different light. Thus, we used Preview annotation tools on a Mac computer to code PDFs of the transcripts. We began with three broad categories: (1) environmental factors; (2) individual factors; and (3) key components of or thoughts about aging, health, and the future. Within the broad category of the environment, we identified factors related to the home, neighborhood, and community, as well as social attitudes. Within individual factors, we identified narratives that indicated character and individual attitudes, access to private financial resources and public financial resources, employment, and advocacy. Within the final group, we identified attitudes and experiences related to hobbies and activities, health challenges and concerns, ability and functioning, exercise, and diet, plans for the future, COVID-19, and understandings of health and healthy aging.

Paraphrases or brief quotes related to these codes were placed in a table, organized by focus group or participant. The content was read, reviewed, and discussed several times. All related excerpts were pulled from the transcripts into one document and then—because there were only 24 participants and the related content had been narrowed—printed and cut into individual quotes. These were placed by hand into codes and broader categories, grouped, and re-grouped. Various overarching themes were identified and considered in light of the existing literature and excerpts read again. Through this iterative process, we identified the role of time, it’s control of the body, and the individual’s control of time. We determined this to be an area receiving limited attention in the literature and worthy of attention.

## Appendix B.

### Appendix B.1. Interview and Focus Group Guide

#### Community Environment

1. Just to start, let’s go around and state where is your favorite place to frequent in your community, and why?
2. In the telephone survey many participants said that their libraries; religious institutions; and shopping malls were accessible;
  - What features of these places make them accessible?
3. In the telephone survey many participants said their grocery stores; restaurants; public parks/rec areas; hotels; were less accessible;
  - How are these places less accessible?

#### Facilitators and Nice Features

1. What features in your community make it easy for you to get around and participate in day to day life?
  - Probe for specific supports/enablers if not mentioned (ramps, curbcuts, disabled parking, community centers, CILs)
  - Usability of public restrooms
    - In telephone survey we found that the accessibility/usability of public restrooms helped 20% of people; limited 40% of people; and for 40% of people had no effect; what is it about public restrooms that make them easy/difficult to use
  - Usability of doctors offices
    - In telephone survey we found that these were relatively accessible for most, but not all people.
    - What is it about doctors offices that make it easy/hard for you to maintain your health—how has this changed with time/age.
  - Link these discussions to the impact on health and healthy aging
    - How do these impact your health behaviors and your health.

#### Barriers Encountered

1. What features in your community make it difficult for you to get around and participate in your everyday life?
  - Tell us about a time it was difficult to get around in your community. What did you do about this barrier? How did you feel?
  - What concerns do you have about barriers to accessibility in your community?
  - Probe for specific barriers if not mentioned (sidewalks, entrances, bathrooms, inside stores)

#### Transportation

1. How does transportation play a role in your ability to engage in your community and to access health care?
  - Probe for modes of transportation, use/access of these modes
  - Has this changed over time?
2. For those who use public transit, who do you encounter when on public transportation? Do they make it easier/harder for you? How? (probe various aspects)
  - a. For those who don’t use public transit, why not?

#### Climate

1. Does weather play a role in your ability to participate in day to day life?
  - Probe for specific examples of weather’s effect on mobility

#### Mobility Devices

1. When do you use your mobility device? Are there certain environments where you use it or where you don’t?
  - For those who use different devices, what determines when you use which device and when?

#### Change over Time

1. Looking ahead, how do you see your personal needs changing as you get older?
  - How about environmental challenges changing as you age?
2. Have you recently changed anything in your daily routine to make it easier to move around your community or home?
  - Has anything gotten more difficult?
3. Thinking about the future, do you think you might need to make any changes in how you move around in your community in the coming years? Particularly as you get older?

#### COVID-19 Question

1. What changes (if any) have you made to your daily routines to maintain wellness during the pandemic?
  - Probe: Caretakers, access to healthcare/food/supplies, transportation
  - Have you been able to access your doctor’s office as frequently as needed?

#### Closing Question

1. Are there any other thoughts you have about growing old or experiences with healthy aging that we haven’t covered today?
